# Retraction Note: Activation of PERK-Nrf2 oncogenic signaling promotes Mdm2-mediated Rb degradation in persistently infected HCV culture

**DOI:** 10.1038/s41598-021-97265-9

**Published:** 2021-09-01

**Authors:** Yucel Aydin, Milad Chedid, Srinivas Chava, Donkita Danielle Williams, Shuanghu Liu, Curt H. Hagedorn, Suchitra Sumitran-Holgersson, Krzysztof Reiss, Krzysztof Moroz, Hua Lu, Luis A. Balart, Srikanta Dash

**Affiliations:** 1 Department of Pathology and Laboratory Medicine, New Orleans, Louisiana USA; 2 Department of Medicine, Division of Gastroenterology and Hepatology, New Orleans, Louisiana USA; 3 Department of Biochemistry, Tulane University Health Sciences Center, New Orleans, Louisiana USA; 4 Department of Medicinal Chemistry, College of Pharmacy, University of Utah, Salt Lake City, UT USA; 5 Department of Medicine and Genetics, University of Arkansas for Medical Sciences, Little Rock, Arkansas USA; 6 Laboratory of Transplantation Surgery and Regenerative Medicine, University of Gothenburg, Gothenburg, Sweden; 7 School of Medicine, LSU Health Sciences Center, New Orleans, Louisiana USA

Retraction of: *Scientific Reports* 10.1038/s41598-017-10087-6, published online 23 August 2017

The Editors have retracted this Article. Concerns were raised regarding a number of figures, specifically:

- Figure 2: the background of some of the blots appear to be unexpectedly clean.
- Figure 9A: the Core panels for TUDCA and PERK inhibitor appear to partially overlap.

The authors have stated they were no longer able to locate the original data. The Editors therefore no longer have confidence in the reliability of the data reported in the article.

Srikanta Dash does not agree with this retraction. Yucel Aydin, Milad Chedid, Srinivas Chava, Donkita Danielle Williams, Shuanghu Liu, Curt H. Hagedorn, Suchitra Sumitran-Holgersson, Krzysztof Reiss, Krzysztof Moroz, Hua Lu, Luis A. Balart have not responded to correspondence regarding this retraction.

